# Deep-learning reconstruction for noncontrast head computed tomography: Improved image quality and potential diagnostic impact in acute ischemic stroke

**DOI:** 10.1007/s00234-026-03955-4

**Published:** 2026-03-21

**Authors:** Dahee Kim, Inpyeong Hwang, Kyu Sung Choi, Heeun Moon, Taehyuk Ham, Young Hun Jeon, Ji Ye Lee, Roh-Eul Yoo, Koung Mi Kang, Tae Jin Yun, Seung Hong Choi, Ji-hoon Kim, Chul-ho Sohn

**Affiliations:** 1 Department of Radiology, Seoul National University Hospital, Seoul, Korea, Republic of; 2 Department of Radiology, College of Medicine, Seoul National University, Seoul, Korea, Republic of

**Keywords:** Head computed tomography, Ischemic stroke, Deep learning, Image reconstruction, Image quality

## Abstract

**Purpose:**

Deep learning reconstruction is a promising technique for improving head CT image quality compared to traditional methods. The purpose of this study was to compare image quality and the potential diagnostic impact of vendor-agnostic deep-learning reconstruction (DLR) versus hybrid iterative reconstruction (IR) for the evaluation of acute ischemic stroke on noncontrast head computed tomography (CT).

**Methods:**

This single-institution retrospective study included 100 patients (mean age 69.6 years; 44 women) who visited the emergency department with suspected acute stroke and underwent noncontrast head CT between November 2021 and November 2022. Fifty patients were confirmed to have acute infarction lesions via subsequent DWI, while the remaining 50 had negative DWI results. The CT images were reconstructed with both IR and DLR. Four reviewers (two radiology residents and two neuroradiologists) assessed subjective image quality and the conspicuity of acute ischemic lesions on a 5-point scale. Nonparametric comparisons of scores and ROC curve analysis were performed.

**Results:**

Compared with IR, DLR resulted in superior overall image quality (4.02 vs. 3.23, *p* < 0.01). While no significant differences were found in lesion conspicuity according to one neuroradiologist (3.90 vs. 3.70; *p* = 0.14), other experienced readers reported significantly enhanced conspicuity with DLR(3.60 vs. 3.12, *p* = 0.014; 3.84 vs. 3.12, *p* < 0.01; and 4.06 vs. 3.62, *p* = 0.018, respectively). ROC curve analysis revealed an increase in the area under the curve (AUC) for radiology resident readers (0.888 vs. 0.844, *p* = 0.002) and a decrease in AUC for neuroradiologists (0.857 vs. 0.911, *p* = 0.034).

**Conclusion:**

Our study demonstrates that DLR offers subjective improvements in image quality, tissue differentiation, and noise reduction. We observed improvement in the diagnostic performance of less experienced radiologists with DLR .

**Supplementary Information:**

The online version contains supplementary material available at 10.1007/s00234-026-03955-4.

## Introduction

Noncontrast head computed tomography (CT) is a first-line diagnostic tool for the evaluation of patients with suspected stroke because of its fast speed, wide availability and feasibility [1, 2]. Early CT signs, such as hypoattenuation compared to healthy brain parenchyma and the absence of a gray matter/white matter (GM/WM) interface, are well recognized for identifying infarcted lesions in ischemic stroke patients. However, the changes can be subtle in early stroke [3, 4]. Furthermore, noncontrast images have intrinsically limited soft tissue contrast resolution [5]. Therefore, improvements in overall image quality and image noise reduction are important factors for the detection of early subtle changes in Hounsfield units (HUs) seen in CT images of ischemic stroke conditions. Previous studies have attempted to improve the overall image quality of head CT by using image reconstruction techniques, such as iterative reconstruction (IR) [6, 7].

Compared with statistics-based reconstruction methods, the reconstruction method using deep learning, which is a subset of machine learning and artificial intelligence, has shown potential for improving image quality in CT [8, 9]. Additionally, previous studies have explored the use of deep-learning reconstruction (DLR) for CT images, revealing its potential to improve diagnostic accuracy and overall image quality [10]. Nevertheless, only a limited number of studies have reported the diagnostic performance of deep learning-reconstructed images for brain CT in clinical settings, including studies on DLR for CT for evaluation of head trauma [11] and acute ischemic stroke [12, 13].

Thus, the purpose of this study was to compare image quality and the potential diagnostic impact of vendor-agnostic DLR versus hybrid IR for the evaluation of acute ischemic stroke on noncontrast head CT.

## Methods

### Subjects

This retrospective study was conducted at a single tertiary center (Seoul National University Hospital, Republic of Korea), and the requirement for written informed consent was waived (IRB No: 2307-207-1457).

**Fig. 1:**
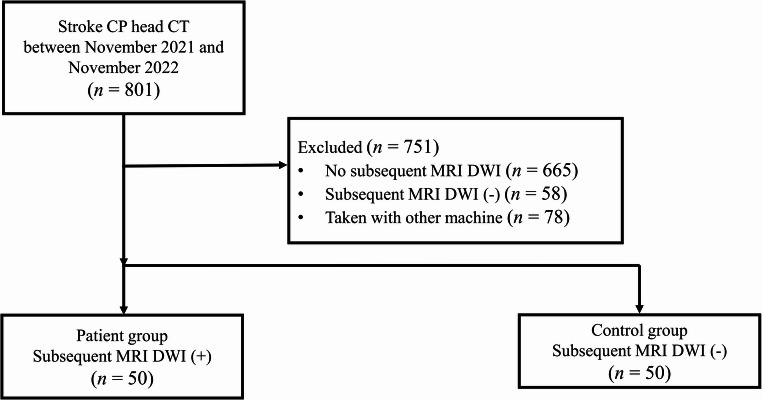
Flowchart of the inclusion and exclusion criteria for the main study population *CP*: Critical pathway; *MRI*: Magnetic resonance imaging; *DWI*: Diffusion weighted image

One radiologist searched the radiology database of our institution from November 2021 to November 2022 to identify patients who met the study eligibility criteria. The inclusion criteria were as follows: (a) patients aged 19 years or older who presented to our institution’s emergency department with suspected acute stroke; (b) patients who underwent noncontrast head CT on a dedicated CT scanner with wide-detector capability for whole-brain perfusion imaging as part of a stroke critical pathway; and (c) patients who underwent subsequent MRI with diffusion-weighted imaging (DWI) within 1 day (Fig. 1). Patients with substantial brain lesions other than ischemic stroke (stroke mimickers, e.g., hemorrhage or tumor) were excluded. The patient group was composed of 50 consecutive patients confirmed to have acute ischemic strokes on the basis of subsequent DWI. In addition, the control group consisted of the first 50 consecutive patients without acute ischemic lesion on DWI. Finally, 100 patients were included in this study. Patient characteristics, including sex, age, last recorded normal time and first recorded abnormal time, were collected from electronic medical records.

### CT acquisition and image reconstruction

All patients underwent noncontrast head CT scans on a 320-channel CT scanner (Aquilion ONE, Canon Medical Systems, Tochigi, Japan). The scan parameters were as follows: tube voltage, 120 kVP; effective tube current time product, 240 mAs; beam collimation, 0.5 × 40 mm; volume computed tomography dose index (CTDIvol), 48.11 mGy; field of view, 240 × 240 mm; and matrix 512 × 512.

**Fig. 2:**
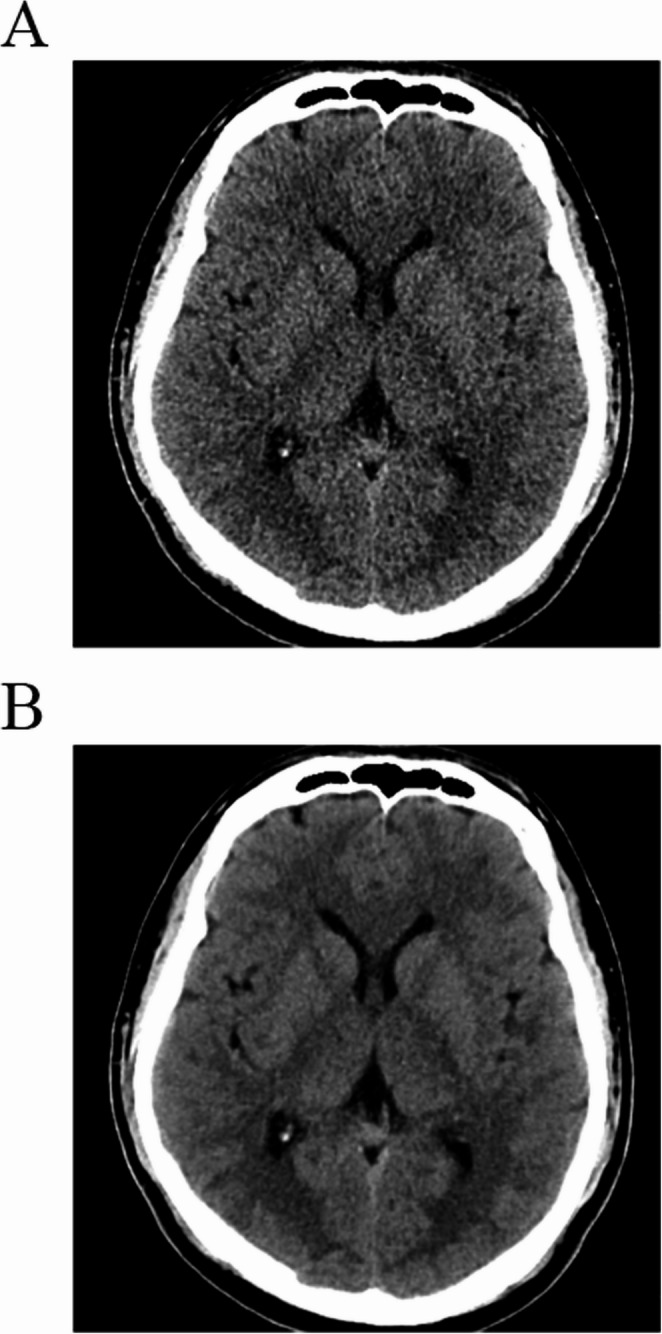
Example of two differently reconstructed normal head images from the same CT scan. Compared to vendor-provided hybrid iterative reconstruction image (adaptive iterative dose reduction, AIDR, Canon Medical Systems) (**a**), deep-learning reconstruction image (ClariCT.AI, ClariPi) exhibited reduced noise and improved delineation of gray-white matter boundaries (**b**)

All CT datasets were reconstructed using vendor-provided hybrid IR (adaptive iterative dose reduction, AIDR) images with brain kernel (FC68) and vendor-agnostic DLR (ClariCT.AI, ClariPi, South Korea) images. The DLR model is a vendor-agnostic U-net–based CNN denoising algorithm trained on > 1 million CT images generated using synthetic sinogram-based low-dose simulation across diverse vendors, scanners, and reconstruction settings. Its performance has been validated in multiple clinical studies of body and pediatric imaging [14–16]. The reconstructed images had a slice thickness of 3 mm and the same field of view. All the images were successfully processed. The representative pair images of normal head CT reconstructed by DLR and IR are shown in Fig. 2.

### Observer study and quantitative measurements

The image quality of the 200 reconstructed noncontrast head CT images was evaluated by two radiology residents (readers 1 and 2, third and second years of training, respectively) and two neuroradiologists (readers 3 and 4, with 12 and 10 years of experience in neuroradiology, respectively). All observers were blinded to the patient information and reconstruction parameters during the evaluation process. The observers could adjust the window width and window level as they deemed appropriate. The observers were blinded to the reconstruction techniques and diagnosis. The images were presented to the observers in randomized order.

Subjective image quality was evaluated using a 5-point Likert scale, assessing both overall image quality score and GM/WM differentiation score. For overall image quality, the scores were as follows. Score 1, nondiagnostic; 2, poor image quality, insufficient for the evaluation of subtle pathology; 3, moderate image quality, sufficient for soft tissue evaluation; 4, good image quality, equal to the current standard; 5, excellent image quality, superior to the current standard. For GM/WM differentiation, the scores were as follows. Score 1, poor GM/WM differentiation, impaired diagnostic quality; 2, reduced GM/WM differentiation, reduced diagnostic quality; 3, acceptable GM/WM differentiation, lower than the current standard; 4, average GM/WM differentiation, equal to the current standard; and 5, better GM/WM differentiation than the current standard. Additionally, the degree of image noise and artifacts was evaluated using a 3-point scale (Supplementary Tables 1 and 2).

Diagnostic performance was assessed through acute ischemic lesion conspicuity using a 5-point scale, and the score parameters were as follows: 1, no focal lesion; 2, low probability (> 5% and < 25%); 3, intermediate probability (> 25% and < 75%); 4, high probability (> 75% and < 90%); and 5, definite (> 90%) (Supplementary Table 3).

### Statistical analysis

For qualitative image quality assessment, mean scores for overall image quality, GM/WM differentiation, image noise and degree of artifacts were calculated for each reader and averaged across all the readers. The mean values were compared using the Wilcoxon signed-rank test. Interobserver agreement was assessed using Gwet’s AC2 with quadratic weights [17]. For diagnostic performance assessment, the mean scores of ischemic lesion conspicuity for each reader were calculated and compared using the Wilcoxon signed-rank test. Single-reader receiver operating characteristic (ROC) curve analyses were conducted for each reader, and multireader, multicase ROC curve analysis with random case and random reader models was performed for the reader subgroups according to the readers’ experience (residents vs. neuroradiologists) [18, 19]. The significance level was set at 0.05. All statistical analyses were performed using R (version 4.2.3; R Core Team, 2023); the “MRMCaov” package was used for multireader ROC analysis, and the “irrCAC” package was utilized for interobserver agreement.

## Results

### Patient groups

A total of 100 patients were included in the study; the 50 patients in the study group consisted of 28 men and 22 women with a mean age of 69.6 years (range, 21–91 years), while the 50 patients in the control group consisted of 28 men and 22 women with a mean age of 68.8 years (range, 28–93 years). The average time intervals between image acquisition and the last recorded normal time and between image acquisition and the first recorded abnormal time were 11 h and 6 h 20 min, respectively. The median infarct volume on subsequent DWI in the study group was 13.4 mL (interquartile range, 3.1-44.8 mL) The mean CTDIvol was 45.4 mGy, and the mean dose-length product was 726.4 mGy∙cm.

### Qualitative image quality analysis

In terms of overall image quality, all the readers reported significantly higher average scores for DLR than for IR (4.02 vs. 3.23, *p* < 0.001). The average scores for GM/WM differentiation and image noise were also significantly greater for DLR than for IR among all the readers (4.07 vs. 3.34, *p* < 0.001; 2.94 vs. 2.25, *p* < 0.001, respectively). In terms of artifacts, the average artifact score was lower in DLR than in IR, indicating that DLR may accentuate the presence of artifacts (2.59 vs. 2.71, *p* = 0.02, respectively.) Detailed observer scores for both DLR and IR are shown in Table 1.

**Table 1:** Detailed observers mean scores of overall image quality, gray/white matter differentiation, noise and artifacts according to the deep-learning reconstruction (DLR) and iterative reconstruction (IR) images

	DLR	IR	*p* value
Overall image quality (5-point Likert Scale)			
Average score of all readers	4.02	3.23	< 0.001
Reader 1	4.12	3.14	< 0.001
Reader 2	4.06	3.26	< 0.001
Reader 3	4.07	3.24	< 0.001
Reader 4	3.82	3.27	< 0.001
**Gray-/white-matter differentiation (5-point Likert Scale)**			
Average score of all readers	4.07	3.34	< 0.001
Reader 1	4.21	3.36	< 0.001
Reader 2	3.97	3.21	< 0.001
Reader 3	4.20	3.38	< 0.001
Reader 4	3.88	3.42	< 0.001
**Noise (3-point Likert Scale**,** higher is better)**			
Average score of all readers	2.94	2.25	< 0.001
Reader 1	2.95	2.02	< 0.001
Reader 2	2.88	2.43	< 0.001
Reader 3	2.95	2.05	< 0.001
Reader 4	2.99	2.51	< 0.001
**Artifacts (3-point Likert Scale**,** higher is better)**			
Average score of all readers	2.59	2.71	0.02
Reader 1	2.45	2.55	0.14
Reader 2	2.68	2.77	0.01
Reader 3	2.43	2.62	0.01
Reader 4	2.78	2.89	0.17

DLR: deep learning reconstruction, IR: iterative reconstruction

### Comparison of lesion conspicuity scores between the patient group and control group

In the patient group, the average scores of lesion conspicuity were significantly greater for DLR than for IR across all the readers (3.60 vs. 3.12, 3.84 vs. 3.12, 3.90 vs. 3.70, and 4.06 vs. 3.62, respectively), with three of the readers (two radiology residents and one neuroradiologist) showing statistically significant differences (*p* = 0.014, < 0.001, 0.137, and 0.017, respectively). With respect to these readers, the proportions of lesion conspicuity scores greater than 4 (indicating a lesion possibility > 75%) increased by 10 percentage points for reader 1, 30 percentage points for reader 2, and 18 percentage points for reader 4.

In the control group, the scores of lesion conspicuity for all the readers were significantly greater for DLR than for IR (1.56 vs. 1.22, 1.46 vs. 1.20, 1.78 vs. 1.34, and 2.08 vs. 1.42, respectively; *p* = 0.009, 0.016, 0.009, and 0.002, respectively.) For all the readers, the proportion of conspicuity score 1 was reduced for DLR. Interestingly, the proportion of scores exceeding 4 (indicating a lesion possibility > 75%) increased among neuroradiologists by 8 percentage points for reader 3, and 16 percentage points for reader 4. Scores of each lesion conspicuity across the patient and control groups as observed by all the readers are also presented in Table 2 and Supplementary Fig. 1.

**Table 2:** Lesion conspicuity scores across the patient group and control group

	Infarct Patient Group (*n* = 50)							Control Group (*n* = 50)						
Score^*^	1	2	3	4	5	Mean	P-value	1	2	3	4	5	Mean	P-value
Reader1 IR	14 (28%)	2 (4%)	9 (18%)	14 (28%)	11 (22%)	3.12	0.014	41 (82%)	7 (14%)	2 (4%)	0 (0%)	0 (0%)	1.22	0.009
Reader1 DLR	6 (12%)	5 (10%)	9 (18%)	13 (26%)	17 (34%)	3.60		31 (62%)	11 (22%)	7 (14%)	1 (2%)	0 (0%)	1.56	
Reader2 IR	11 (22%)	3 (6%)	16 (32%)	9 (18%)	11 (22%)	3.12	< 0.001	41 (82%)	8 (16%)	1 (2%)	0 (0%)	0 (0%)	1.20	0.016
Reader2 DLR	6 (12%)	0 (0%)	9 (18%)	16 (32%)	19 (38%)	3.84		35 (70%)	8 (16%)	6 (12%)	1 (2%)	0 (0%)	1.46	
Reader3 IR	5 (10%)	0 (0%)	11 (22%)	23 (46%)	11 (22%)	3.70	0.137	38 (76%)	7 (14%)	5 (10%)	0 (0%)	0 (0%)	1.34	0.009
Reader3 DLR	5 (10%)	3 (6%)	6 (12%)	14 (28%)	22 (44%)	3.90		28 (56%)	9 (18%)	9 (18%)	4 (8%)	0 (0%)	1.78	
Reader4 IR	5 (10%)	0 (0%)	19 (38%)	11 (22%)	15 (30%)	3.62	0.017	39 (78%)	4 (8%)	4 (8%)	3 (6%)	0 (0%)	1.42	0.002
Reader4 DLR	5 (10%)	1 (2%)	9 (18%)	6 (12%)	29 (58%)	4.06		27 (54%)	5 (10%)	7 (14%)	9 (18%)	2 (4%)	2.08	

DLR: deep learning reconstruction, IR: iterative reconstruction. Numbers in the parentheses are percentages

^*^Lesion conspicuity scores were as follows: 1, no focal lesion; 2, low probability (> 5% and < 25%); 3, intermediate probability (> 25% and < 75%); 4, high probability (> 75% and < 90%); and 5, definite (> 90%)

### Diagnostic performance for stroke lesion detection

**Fig. 3:**
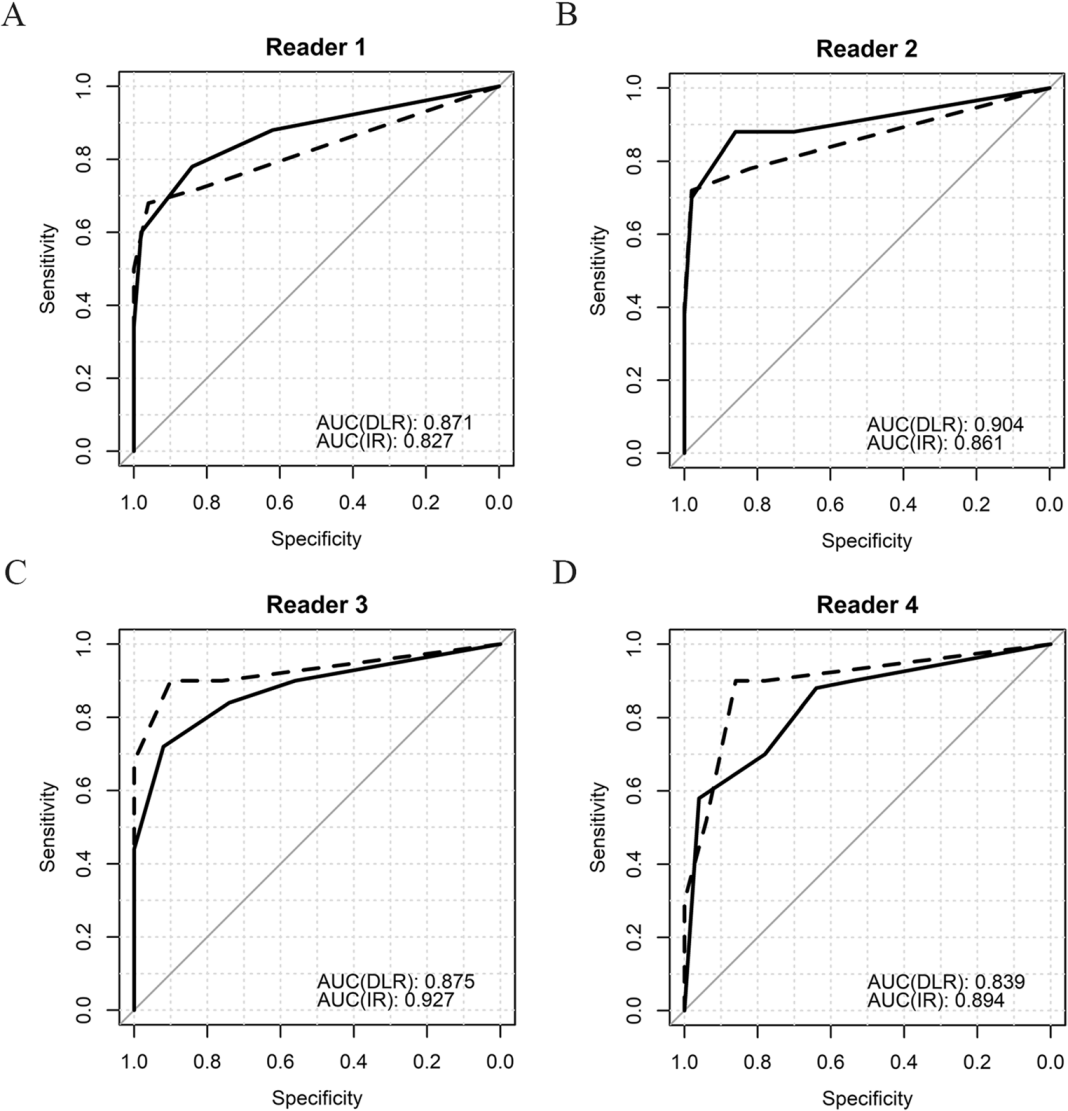
Receiver operating characteristic curves of each reader. Thick lines represent deep learning reconstruction image (DLR)-based curves, and dotted lines represent iterative reconstruction image (IR)-based curves *DLR*: Deep-learning reconstruction image; *IR*: Iterative reconstruction image; *AUC*: Area under the curve; *CI*: Confidence interval

**Fig. 4:**
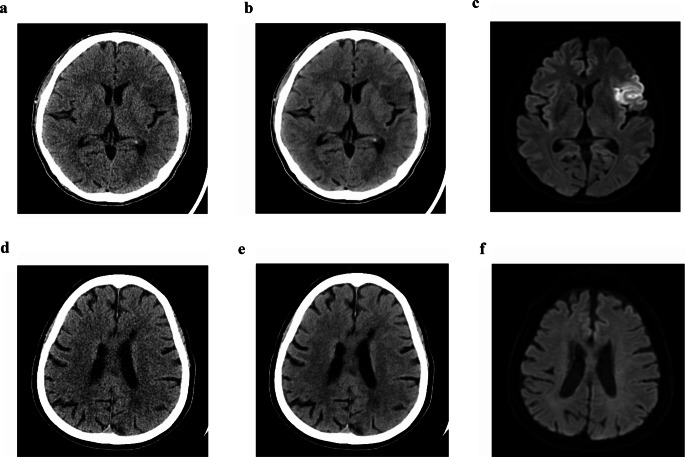
Representative cases. Case 1: 60-year-old male with acute left middle cerebral artery superior division infarction. Iterative reconstruction (IR) (**a**) and deep-learning reconstruction (DLR) (**b**) images are shown pairwise with corresponding diffusion-weighted imaging (DWI) (**c**) acquired within 30 min of CT. Two resident readers scored IR as 1 (no acute lesion), DLR as 3 and 4 (intermediate, high probability). Neuroradiologists scored IR 3, 4 and DLR 4. Case 2: 79-year-old female presented with dysarthria (onset 4.5 h). IR (**d**) and DLR (**e**) images are shown; DWI obtained within 30 min (**f**) and follow-up DWI at 4 days (not shown) revealed no acute ischemia-infarction lesion. All readers scored IR as 1 (no acute lesion). DLR scores were higher: one neuroradiologist scored 4 (high probability), others scored 3 (intermediate probability), likely due to accentuated chronic ischemic changes on DLR

The diagnostic performance of DLR and IR was assessed using ROC curve analyses. In the single-reader analysis, there was no statistically significant difference for each reader (all *p* > 0.05; Table 3). The area under the ROC curve (AUC) values for the radiology residents were greater for DLR than for IR (0.888 vs. 0.844; *p* = 0.002), whereas those for the neuroradiologists were lower for DLR than for IR (0.857 vs. 0.911; *p* = 0.034). The ROC curves of each reader are shown in Fig. 3. Representative case of acute ischemic stroke patients and potential false-positive case in controls are illustrated in Fig. 4.

**Table 3:** Receiver operating characteristic (ROC) curve analysis for the diagnosis of acute ischemic lesions for deep-learning reconstruction (DLR) and iterative reconstruction (IR) images

	AUC (DLR)	AUC (IR)	*p* value
Reader 1	0.871 (0.802–0.941)	0.827 (0.751–0.903)	0.24
Reader 2	0.904 (0.843–0.966)	0.861 (0.791–0.931)	0.094
Reader 3	0.875 (0.807–0.944)	0.927 (0.874–0.980)	0.054
Reader 4	0.839 (0.762–0.916)	0.894 (0.832–0.957)	0.082
Radiology residents^*^	0.888 (0.821–0.955)	0.844 (0.774–0.914)	0.002^†^
Neuroradiologists^*^	0.857 (0.776–0.939)	0.911 (0.843–0.979)	0.034^†^

^*^ Multireader, multicase ROC analysis for each reader group

^†^*p* < 0.05

Data in parentheses are 95% confidence intervals. AUC: area under the ROC curve, DLR: deep-learning reconstruction; IR: iterative reconstruction

### Interobserver agreement

Gwet’s AC2 values for overall image quality, GM/WM differentiation, image noise, and artifact scores were 0.930 (95% CI, 0.919–0.942), 0.912 (95% CI, 0.899–0.925), 0.886 (95% CI, 0.864–0.908), and 0.885 (95% CI, 0.861–0.909), respectively.

## Discussion

Head CT image quality has been continuously improved through techniques like IR [6, 7]. DLR represents a recent advancement with promising improvements in image quality; however, its diagnostic impact in clinical head CT, especially for acute ischemic stroke, remains under-explored [11–13]. In our study, we compared the qualitative image quality and diagnostic performance of vendor-agnostic DL-based image reconstruction (DLR) and vendor-provided hybrid iterative reconstruction (IR) for noncontrast head CT in the context of detecting acute ischemic stroke lesions. DLR resulted in superior overall image quality (4.02 vs. 3.23), increased GM/WM differentiation (4.07 vs. 3.34), and effective noise reduction (2.94 vs. 2.25). Given the small difference in attenuation between GM/WM in noncontrast head CT [5, 12], the significance of these enhancements becomes particularly pronounced in the context of early stroke detection, for which subtle changes in HUs demand a high level of image clarity and contrast resolution. Our study further evaluated the diagnostic performance of both reconstruction methods, focusing on the conspicuity of acute ischemic lesions.

These results align with those of prior studies that demonstrated the ability of DLR to enhance image quality and reduce noise in various medical imaging modalities, including CT [10, 12, 20–25]. Additionally, a previous meta-analysis comparing two DL reconstruction models (True Fidelity [TF] and Advanced intelligent Clear-IQ Engine [AiCE]) and iterative reconstruction (IR) for abdominal CT revealed the superior ability of DLRs to reduce image noise while preserving desirable image texture [24]. A phantom study of the upper abdomen also revealed the superiority of DLR over IR for lesion detection and dose reduction [25]. In the neuroradiology field, efforts to improve CT image quality have also long been pursued, and DLR represents a recent extension of this trend. Before the introduction of DLR, IR techniques demonstrated clear benefits for acute stroke imaging by enhancing image quality and yielding higher interobserver agreement for Alberta stroke program early CT score scoring with better correspondence to the final infarct extent [26, 27]. Although there have been few prior studies of DLR in head CT [12, 13, 28], recent evidence that these improvements translate to superior detection of acute ischemic changes remains limited. Our study demonstrated the potential impact of DLR on stroke diagnosis on noncontrast head CT, particularly by enhancing the performance of less experienced readers.

Interestingly, while experienced readers did not exhibit significant differences in lesion conspicuity between DLR and IR, less experienced readers demonstrated a clear advantage using DLR. These findings suggest that the benefits of DLR may be more pronounced for radiologists with less experience, potentially aiding in the early identification of ischemic stroke lesions. The improved performance observed by less experienced readers could be attributed to the advanced ability of DLR to enhance the delineation of subtle structures, contributing to better lesion conspicuity and diagnostic accuracy. In contrast, the application of DLR in the control group resulted in a reduction in score of 1 across all readers, indicating a decreased proportion of cases classified as having no stroke possibility. This effect was particularly pronounced among neuroradiologists, who demonstrated a decrease in AUC values. We hypothesize that these findings may be attributable to the increased conspicuity of chronic ischemic lesions visible with DLR, leading to an overestimation of acute stroke probability. Therefore, we recommend that experienced readers utilizing DLR images for interpretation concurrently evaluate conventional IR images to maintain diagnostic rigor and to avoid potential misinterpretation based on subtle findings suggestive of chronic pathology.

Although our study could not evaluate improvements in terms of artifacts, we expect that the results suggest enhancements in the delineation of subtle structures using DLR [22], thereby improving artifact detection. The results of the diagnostic performances of neuroradiologists, evaluated with lesion conspicuity using ROC curve analysis, also suggest that improved delineation of subtle, low-attenuated structures in noncontrast head CTs led to an increased false-positive rate in the control group of neuroradiologists, especially in the reader who had more clinical experience (reader 4). Additionally, as we did not provide clinical information or symptoms of the patients (such as lateralization signs, which strongly suggest stroke or a previous history of ischemic stroke) in this study, the detection of chronic ischemic lesions, which also presented as low-attenuated lesions, would have increased in the control group. In addition, we did not evaluate whether the predicted infarct location matched the true lesion, nor did we assess individual early ischemic signs on CT. Therefore, our study did not fully represent real-world clinical practice. However, the scope of our retrospective study was designed to explore overall image quality and its impact on diagnostic performance. Thus, further prospective studies are warranted to prove the benefits of DLR in real acute stroke clinical settings.

GM/WM differentiation scores were significantly higher for DLR images compared to IR images across all readers (4.07 vs. 3.34). A score of 4 was defined as “average GM/WM differentiation, equal to the current standards.”, reflecting the image quality typically observed in standard CT examinations performed on patients without acute pathology or significant imaging artifacts. While the lower scores observed for IR might suggest inferior quality than standards, it is important to consider the study population: patients suspected of acute ischemic stroke prone to reduced cooperation and involuntary motion that can compromise image acquisition. A previous report has demonstrated frequent motion artifacts in CT scans of unconscious emergency department patients [29]. Despite these potentially unfavorable imaging conditions, DLR maintained scores approximating the standard score. These findings suggest that DLR would help maintain GM/WM differentiation even in the presence of common degradations encountered in real-world emergency settings. However, improved GM/WM differentiation with DLR did not correlate with superior diagnostic performance. DLR is known to generate artificial structures due to hallucination, potentially leading to false positives [30]. This was observed in our study where neuroradiologists reported an increased rate of false positive findings in the control group, necessitating caution regarding its broad application.

This study has several limitations. First, the study is a retrospective study with a relatively small number of enrolled patients. Second, this study is a single-center and single-vendor study, and selection bias may exist. Therefore, to confirm the study results, further multicenter and multivendor studies involving a greater number of patients are needed. Additionally, the absence of an evaluation encompassing stroke mimics, such as intracranial hemorrhage, requires further investigation to define the full scope of the diagnostic performance of DLR. Finally, owing to the retrospective nature of the study, radiation dose reduction in head CT was not addressed in this study. Considering that the results of this study revealed the superiority of DLR in terms of overall image quality and noise reduction over IR, radiation dose reduction would be possible by altering the reconstruction mechanism. Further prospective studies evaluating the impact of DLR on reduced radiation dose protocols are needed.

## Conclusions

In conclusion, our study demonstrates that DLR offers subjective improvements in image quality, tissue differentiation, and noise reduction, compared with IR in noncontrast head CT. We observed a potential benefit for less experienced radiologists with DLR in the detection of acute ischemic stroke. However, an increased risk of false positive findings may be observed, which could be more pronounced depending on reader experience. Further large-scale, prospective investigation is warranted to assess the role of DLR in head CT and determine whether it can improve assessment of acute ischemic stroke, particularly when used by less experienced readers.

## Supplementary Information

Below is the link to the electronic supplementary material.

Supplementary Material 1 (DOCX 297 KB)

## Data Availability

The data supporting this article cannot be made publicly available because they include protected patient health information.

## Abbreviations

AIDR: Adaptive iterative dose reduction
CT: Computed tomography
CTDIvol: Volume computed tomography dose index
DLR: Deep-learning reconstruction
DWI: Diffusion-weighted imaging
GM: Gray matter
HUs: Hounsfield units
IR: Iterative reconstruction
ROC: Receiver operating characteristic
WM: White matter
