# Supplementary material for: Deep-learning reconstruction for noncontrast head computed tomography: Improved image quality and potential diagnostic impact in acute ischemic stroke
Source: Neuroradiology. 2026 Mar 21;68(4):965–74. doi: 10.1007/s00234-026-03955-4 (PMC13139282; doi:10.1007/s00234-026-03955-4)
Supplement: Supplementary file 1 — Supplementary Material 1 (DOCX 297 KB) [file 234_2026_3955_MOESM1_ESM.docx]

**Supplementary Materials**

**Supplementary Table 1.** Description of the categories of image quality characteristics (overall image quality and gray/white matter differentiation)

| Image quality characteristic | 1 | 2 | 3 | 4 | 5 |
| --- | --- | --- | --- | --- | --- |
| Overall image quality | Nondiagnostic | Poor image quality, insufficient for the evaluation of subtle pathology | Moderate image quality, sufficient for soft tissue evaluation | Good image quality, equal to the current standard | Excellent image quality, superior to the current standard |
|  |  |  |  |  |  |
| Gray-/white-matter differentiation | Poor GM/WM differentiation, impaired diagnostic quality | Reduced GM/WM differentiation, reduced diagnostic quality | Acceptable GM/WM differentiation, lower than the current standard | Average GM/WM differentiation, equal to the current standard | Better GM/WM differentiation compared to the current standard |

*GM:* gray matter; *WM:* white matter

**Supplementary Table 2.** Description of categories of image quality characteristics (noise and artifacts)

| Image quality characteristic | 1 | 2 | 3 |
| --- | --- | --- | --- |
| Noise | Marked (affecting image interpretation) | Minor | Minimal or none |
|  |  |  |  |
| Artifacts | Marked (affecting image interpretation) | Minor | Minimal or none |

**Supplementary Table 3.** Description of lesion conspicuity categories

| Lesion conspicuity score | 1 | 2 | 3 | 4 | 5 |
| --- | --- | --- | --- | --- | --- |
| Meaning | No focal lesion suggesting acute ischemic stroke | Low probability  (> 5% and < 25%) | Intermediate probability  (> 25% and < 75%) | High probability  (> 75% and < 90%) | Definite  (> 90%) |

**Supplementary Figure 1.** Stacked bar graphs depicting the scores for each lesion conspicuity across the patient group (a) and control group (b).

(a)
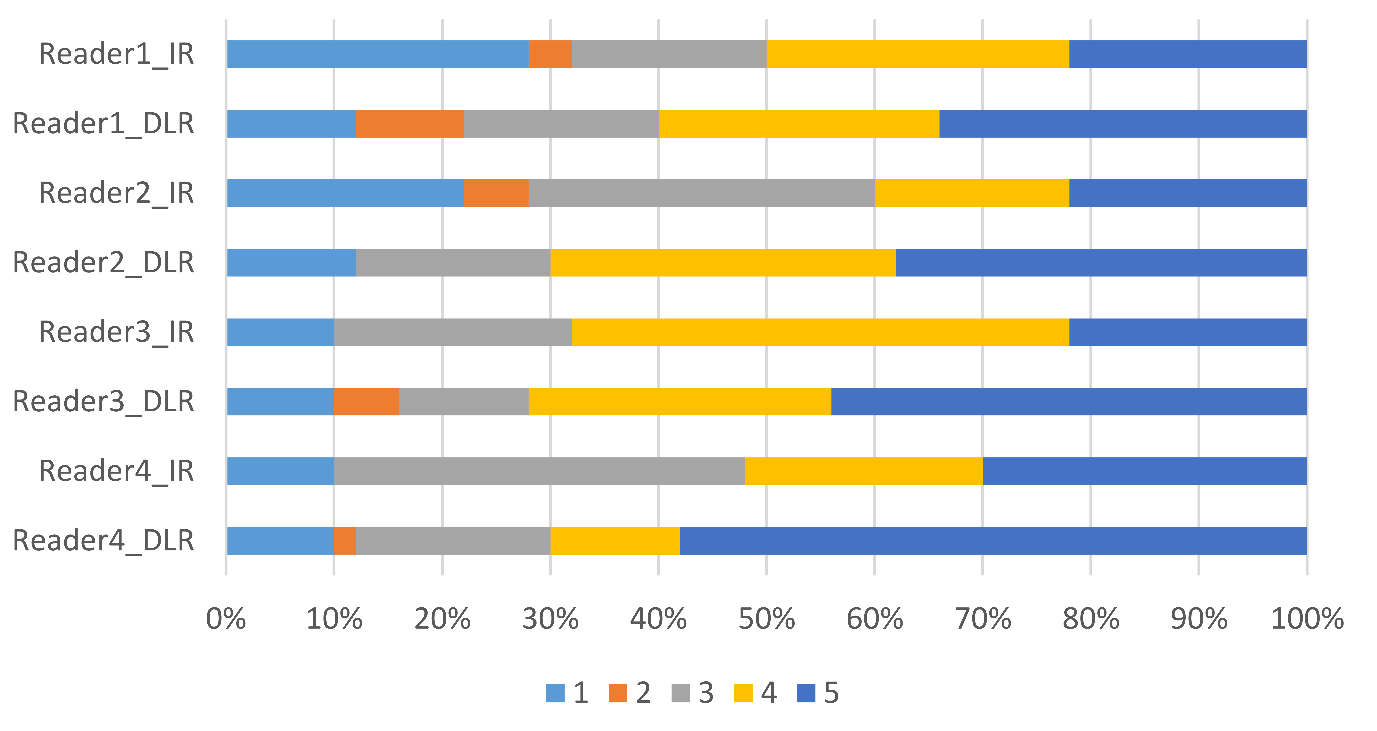


(b)
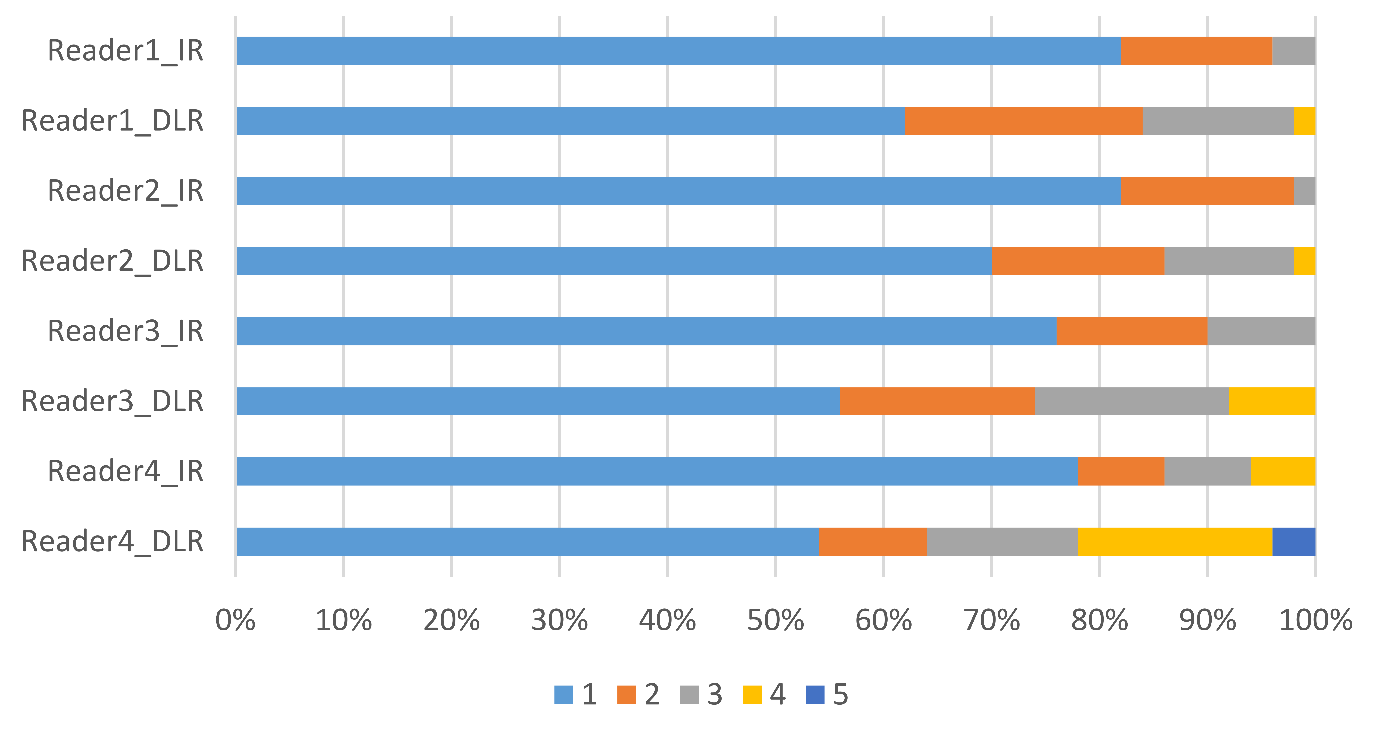


DLR: Deep-learning reconstruction image, IR: Iterative reconstruction image
